# Gas Phase and Gas–Solid Interface Ozonolysis of Nitrogen Containing Alkenes: Nitroalkenes, Enamines, and Nitroenamines

**DOI:** 10.1021/acs.jpca.2c04400

**Published:** 2022-08-04

**Authors:** Weihong Wang, Xinke Wang, Pascale S. J. Lakey, Michael J. Ezell, Manabu Shiraiwa, Barbara J. Finlayson-Pitts

**Affiliations:** Department of Chemistry, University of California, Irvine, California 92697-2025, United States

## Abstract

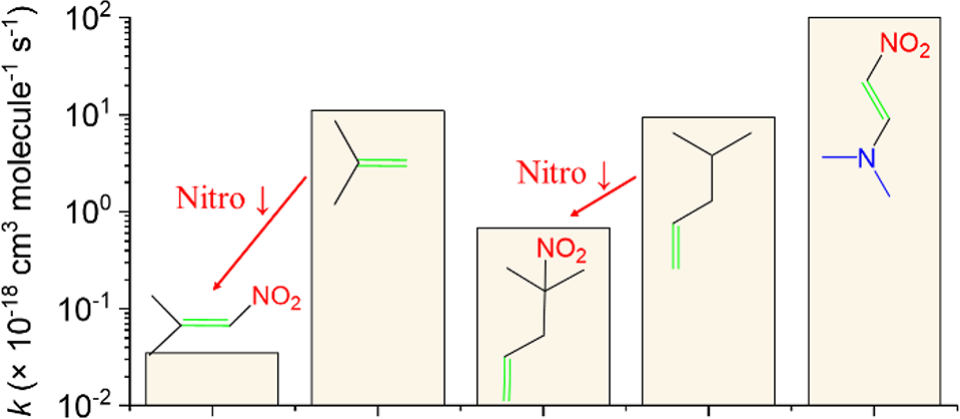

Emerging contaminants are of concern due to their rapidly increasing numbers and potential ecological and human health effects. In this study, the synergistic effects of the presence of multifunctional nitro, amino and carbon–carbon double bond (C=C) groups on the gas phase ozonolysis in O_2_ or at the air/solid interface were investigated using five simple model compounds. The gas phase ozonolysis rate constants at 296 K were (3.5 ± 0.9) × 10^–20^ cm^3^ molecule^–1^ s^–1^ for 2-methyl-1-nitroprop-1-ene and (6.8 ± 0.8) × 10^–19^ cm^3^ molecule^–1^ s^–1^ for 4-methyl-4-nitro-1-pentene, with lifetimes of 134 and 7 days in the presence of 100 ppb ozone in the atmosphere, respectively. The rate constants for gas phase *E*-*N*,*N*-dimethyl-1-propenylamine and *N*,*N*-dimethylallylamine reactions with ozone were too fast (>10^–18^ cm^3^ molecule^–1^ s^–1^) to be measured, implying lifetimes of less than 5 days. A multiphase kinetics model (KM-GAP) was used to probe the gas–solid kinetics of 1-dimethylamino-2-nitroethylene, yielding a rate constant for the surface reaction of 1.8 × 10^–9^ cm^2^ molecule^–1^ s^–1^ and in the bulk 1× 10^–16^ cm^3^ molecule^–1^ s^–1^. These results show that a nitro group attached to the C=C lowers the gas phase rate constant by 2–3 orders of magnitude compared to the simple alkenes, while amino groups have the opposite effect. The presence of both groups provides counterbalancing effects. Products with deleterious health effects including dimethylformamide and formaldehyde were identified by FTIR. The identified products differentiate whether the initial site of ozone attack is C=C and/or the amino group. This study provides a basis for predicting the environmental fates of emerging contaminants and shows that both the toxicity of both the parent compounds and the products should be taken into account in assessing their environmental impacts.

## Introduction

Potential new contaminants are emerging almost every day and have been widely detected in the environment.^1−10^ They can reside in soil and water systems for years and produce adverse ecological and human health effects.^11−13^ There is an urgent need for molecular level insight into their environmental fates since their degradation can form products that are more toxic than the parent compounds. However, studying all environmentally relevant reactions of each compound is impossible due to the large and increasing numbers of emerging contaminants. An alternative approach is to investigate model compounds with different combinations of common functional groups in order to develop an understanding of what controls their reaction kinetics and mechanisms relevant to their environmental degradation processes.

**Figure 1:**
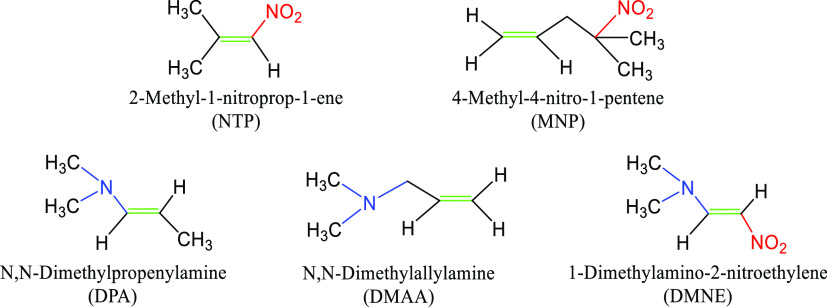
Structures of model compounds.

Nitro, amino, and carbon–carbon double bond (C=C) functional groups are common moieties in emerging contaminants such as pharmaceuticals, pesticides, and munitions. In a separate study, ozonolysis in CCl_4_ of five model compounds with different combinations of these functional groups, i.e., 2-methyl-1-nitroprop-1-ene (NTP), 4-methyl-4-nitro-1-pentene (MNP), *E-N*,*N*-dimethyl-1-propenylamine (DPA), *N*,*N*-dimethylallylamine (DMAA), and 1-dimethylamino-2-nitroethylene (DMNE) (Figure 1), was investigated.^14^ The results showed that a nitro group attached to C=C can dramatically increase the activation energies and decrease rate constants for alkene ozonolysis, while an amino group has the opposite effect. In addition, ozone can attack either the C=C, the amine nitrogen, or both, depending on the different combinations of these moieties in a molecule. While these studies elucidated the relative reactivities and mechanisms in a condensed liquid phase, some of the compounds have sufficiently high vapor pressures to be present in the gas phase, while others remain as a solid. In the absence of a solvent cage that holds reactive fragments together and dampens energy, different kinetics, products, and mechanisms may apply. Hence, defining the chemistry in the gas phase and at the gas–solid interface is also needed for assessing their environmental fates.

In this study, ozonolysis of these five model compounds was investigated in the gas phase and at the gas–solid interface. These data provide important guidance to policy makers and regulators on the environmental fates and potential impacts of some important pharmaceuticals, pesticides, and munitions that are widely used. This is a critical step toward developing more sustainable substitutes.

## Experimental Section

### Materials

The model compounds chosen for these studies are 2-methyl-1-nitroprop-1-ene (NTP, AK Scientific, 95%), 4-methyl-4-nitro-1-pentene (MNP, MuseChem, 96%), *N*,*N*-dimethylallylamine (DMAA, Sigma-Aldrich, ≥99%), and 1-dimethylamino-2-nitroethylene (DMNE, Sigma-Aldrich, 97%) and were used as received. *N*,*N*-Dimethyl-1-propenylamine (DPA) was synthesized by condensation of anhydrous dimethylamine with propionaldehyde following the procedure of Ellenberger et al.^15^ as described in detail elsewhere.^14^ DPA was shown by ^1^H NMR to be the *E* isomer^16^ with a purity of 93% measured using GC–MS. To confirm identification and yields of some products, the ozonolysis of 2,3-dimethyl-2-butene (DMB, Sigma-Aldrich, ≥99%) was also studied; the DMB was used as received. In all cases, the liquids were subjected to several freeze–pump–thaw cycles and the vapor obtained from the headspace at room temperature.

### Gas-Phase Studies by Transmission FTIR

Gas phase studies were carried out in static-mode experiments in a cell with ZnSe windows (Figure S1A). The path length of the cell was 10 cm with a volume of ∼60 cm^3^. The cell was first evacuated and gas phase NTP, MNP, DPA or DMAA added to the cell through a glass manifold. Ozone in O_2_ was generated by photolysis of O_2_ (Praxair, 99.993%) using a low-pressure mercury lamp (UV Products, model D-23017). O_3_/O_2_ mixtures were then expanded into the cell to a total pressure of 760 Torr. In some experiments, cyclohexane (Fisher Scientific, 99.9%) was added as an OH radical scavenger.^17^ Transmission infrared spectra were recorded as a function of time using a Mattson Cygnus FTIR at 1 cm^–1^ resolution and 32 coadded scans.

For kinetics studies, a reaction scheme for NTP or MNP ozonolysis was developed (Tables S1 and S2) and numerical integration carried out using Kintecus.^18^ The NTP-O_3_ (or MNP-O_3_) reaction rate constant, branching ratio, OH radical yield, and the NTP-OH (or MNP-OH) reaction rate constant were varied until the best fit to the NTP (MNP) and O_3_ decays was achieved (Figure S2).

### DMAA-O_3_ Product Studies by DART-MS

Direct analysis in real-time mass spectrometry (DART-MS, Ionsense, DART-SVP with Vapur Interface) coupled to a triple quadrupole mass spectrometer (Waters, Xevo TQS) was used to measure the reaction product of gas phase DMAA ozonolysis. Reactions of DMAA with O_3_ were performed in a 700 mL cylindrical glass cell with inlet and outlet valves at the opposite ends. The cell was first evacuated and DMAA followed by a mixture of O_3_/O_2_ was expanded into the cell. After the reaction, N_2_ gas was flowed through the cell at 210 mL min^–1^ directly into the DART-MS source in positive ion mode. The helium reagent gas flow for DART was 3.1 L min^–1^, the grid electrode voltage was 350 V, and the reagent gas temperature was set at room temperature.

### Gas–Solid DMNE Studies

The nitroenamine DMNE is solid at room temperature. Product studies of DMNE ozonolysis were carried out in the same 10 cm cell used for other gas phase studies. Solid DMNE was first dissolved in acetonitrile (Sigma-Aldrich, ≥99.9%) and a small amount of the solution deposited on the ZnSe windows. A thin film of DMNE was formed as solvent evaporated. The total number of DMNE molecules was in the range of (0.4–4.1) × 10^17^ molecules. The cell was then assembled under vacuum and secured in a custom holder. The O_3_/O_2_ mixture (with O_3_ concentrations of (3.2–8.5) × 10^15^ molecules cm^–3^) was expanded into the cell and transmission FTIR used to probe the combined film on the windows and the gas phase.

For the kinetics studies, changes in solid DMNE were followed using attenuated total reflectance (ATR)-FTIR (Figure S1B). A measured amount of DMNE in acetonitrile solution was applied on an ATR crystal (Ge, 4 mm × 10 mm × 80 mm, 45°, Pike Technologies). As the solvent evaporated, a thin film of DMNE was left on the Ge crystal which was mounted in an ATR holder. The available surface area of the crystal was 4 cm^2^, and the total amount of DMNE deposited varied from (1.6–9.9) × 10^16^ molecules, giving a column concentration of (0.4–2.5) × 10^16^ molecules cm^–2^. The number of monolayers of DMNE on the crystal was calculated using the density and molecular weight of DMNE (1.073 g cm^–3^, MW 116). This is equal to 5.56 × 10^21^ molecules cm^–3^. Assuming that the film was randomly formed and average dimension is the same in all three directions, the number of molecules per cm^2^ for a monolayer (ML) would be (5.56 × 10^21^)^2/3^ cm^–2^ or 3.1 × 10^14^ molecules cm^–2^. From the range of column concentrations on the crystal, the number of monolayers is estimated to be 13–80 with the maximum thickness of ∼0.05 μm. This is much less than the calculated depth of penetration (*d*_p_) of the thin film of 0.52 μm ^19^ at 1236 cm^–1^.

Mixtures of O_3_/O_2_ were further diluted with N_2_ to concentrations in the range (0.34–2.3) × 10^13^ molecules cm^–3^. These mixtures flowed through the ATR cell at 1 L min^–1^, exposing the DMNE thin film to a constant concentration of O_3_. The loss of DMNE at 1236 cm^–1^ which corresponds to the −NO_2_ symmetric stretch was followed as a function of time using a Thermo Nicolet 6700 FTIR at 4 cm^–1^ resolution and 16 coadded scans.

Because the reaction is a multiphase gas–solid reaction, the kinetics of this heterogeneous DMNE ozonolysis were analyzed using a modified version of the kinetic multilayer model KM-GAP.^20,21^ Processes included in KM-GAP are gas-phase diffusion, reversible adsorption to a surface, partitioning into a solid, bulk diffusion, and chemical reactions. More details regarding the KM-GAP modeling of multiphase DMNE ozonolysis are found in Text S1.

## Results and Discussion

### Gas Phase Kinetics

The model compounds have different combinations of −NO_2_, amino, and C=C groups. For nitroalkene ozonolysis, the loss of NTP (or MNP) and ozone was followed by FTIR and the rate constant was derived using numerical integration of the reaction scheme in Tables S1 and S2. The room temperature rate constants were determined to be (3.5 ± 0.9) × 10^–20^ cm^3^ molecule^–1^ s^–1^ for NTP ozonolysis and (6.8 ± 0.8) × 10^–19^ cm^3^ molecule^–1^ s^–1^ for MNP ozonolysis. For DMAA and DPA, the reactions were too fast to be followed by FTIR in these experiments, indicating lower limit rate constants of >10^–18^ cm^3^ molecule^–1^ s^–1^.

The gas phase rate constants are summarized in Table 1. Similar to the ozonolysis in CCl_4_,^14^ the presence of an −NO_2_ group on the double bond in NTP lowers its gas phase rate constant significantly, in this case by 2–3 orders of magnitude compared to that for 2-methylpropene. It is also about a factor of 3 smaller than that for 2-chloro-1-propene which has the electron-withdrawing halogen on the double bond. When the −NO_2_ group is displaced by two carbons from the double bond as in MNP, the rate constant is also lower, by about an order of magnitude, compared to that for the terminal alkenes such as 4-methyl-1-pentene. It is clear that the strong electron withdrawing power of the −NO_2_ group significantly lowers the rate constants for alkene ozonolysis.

**Table 1:**
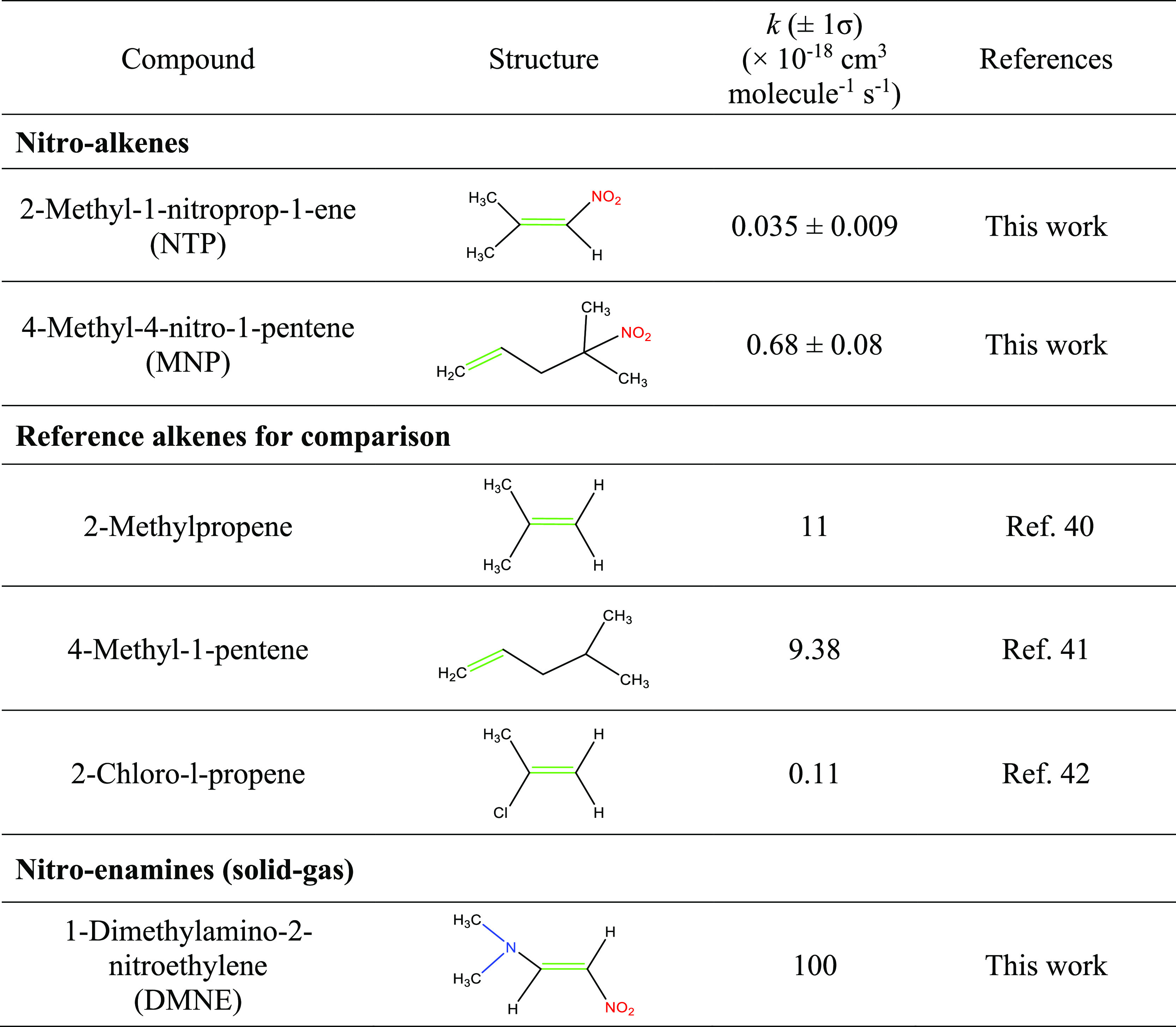
Rate Constants for the Gas Phase or Solid–Gas Ozonolysis of Model Compounds and Selected Alkenes at 296 K

The gas phase rate constants measured here for NTP and MNP are a factor of 3 and 34 times lower, respectively, than measured in CCl_4_ solution.^14^ This difference is consistent with results reported for the ozonolysis of simple alkenes^22^ and chlorinated alkenes^23^ in CCl_4_ compared to those in the gas phase. The initial interaction of ozone with the double bond can form a complex that either decomposes back to reactants or goes on to form products. The presence of a solvent can stabilize the complex against decomposition back to reactants, thus favoring the conversion of the complex to products.^23^

### Gas–Solid Kinetics

In the ATR-FTIR studies, changes in a thin film of DMNE on an ATR crystal were followed in a flow system as a constant concentration of ozone flowed through the cell. A slow loss of solid DMNE due to sublimation was observed even in the absence of O_3_, with the net loss depending on the N_2_ flow rate and the number of DMNE layers (Figure S3). To elucidate and quantify the processes occurring in the absence and presence of O_3_, a multiphase kinetics model, KM-GAP,^20^ was applied. This model takes into account all of the physical (e.g., exchange between the gas and solid phases, diffusion in both phases, etc.) as well as chemical processes that occur on exposure to a reactive gas that result in changes in the composition layer by layer as a function of time. In the present case, the saturation vapor pressure of DMNE, its first order loss from the gas phase to surfaces, and its bulk diffusion coefficient were obtained (Table S3) through the best fits to the DMNE loss in the absence of O_3_ (Figure S3). These parameters were then applied to model the DMNE loss in the presence of O_3_ where transport of O_3_ to the surface and diffusion into the solid as it reacts are taken into account. These change the composition layer by layer as a function of time.

Figure S4 shows the experimentally measured decay of DMNE in the film as a function of time for a variety of O_3_ concentrations and film thicknesses. The rates of decay in the presence of O_3_ are significantly higher than in its absence (Figure S3) where only loss by sublimation occurred. Since no products were detected by FTIR in the film during reaction (but many gas phase products were observed in the static experiments; see below), it was first assumed that only gas phase products were generated. The model predicted complete loss of DMNE (Figure S4) that is inconsistent with the experimental data which reached a plateau at longer reaction times. As seen in Figure S4, including the formation of a nonvolatile product with a yield of 0.06 significantly improved the agreement between the experimental data and the model predictions. Although no products were detected by FTIR, DART-MS measurements of DMNE on a screen and exposed to ozone did show some peaks not attributable to DMNE. Inputs and best fit parameters are summarized in Table S3. The DMNE-O_3_ reaction rate constant in the bulk phase derived from the model is 1.0 × 10^–16^ cm^3^ molecule^–1^ s^–1^, about a factor of 10 slower than that in CCl_4_. The gas–solid rate constant is about 2 orders of magnitude larger than that for the reaction of gas phase O_3_ with a thin film of another atmospherically relevant nitroenamine, the neonicotinoid pesticide nitenpyram (NPM),^21^ for there is greater steric hindrance than for DMNE. This was also reflected in the smaller Arrhenius pre-exponential factor for the rate constant of NPM compared to DMNE in CCl_4_ solutions.^14^

### Gas Phase Product Studies

**Figure 2:**
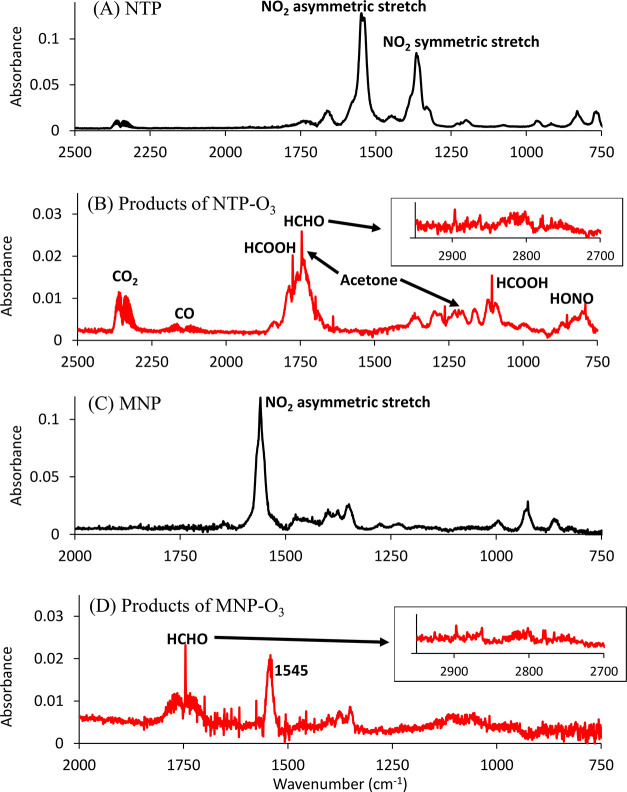
FTIR spectra of (A) NTP, (B) products of NTP ozonolysis after subtraction of the signal from excess NTP, (C) MNP, and (D) products of MNP ozonolysis after subtraction of the signal from excess MNP.

**Figure 3:**
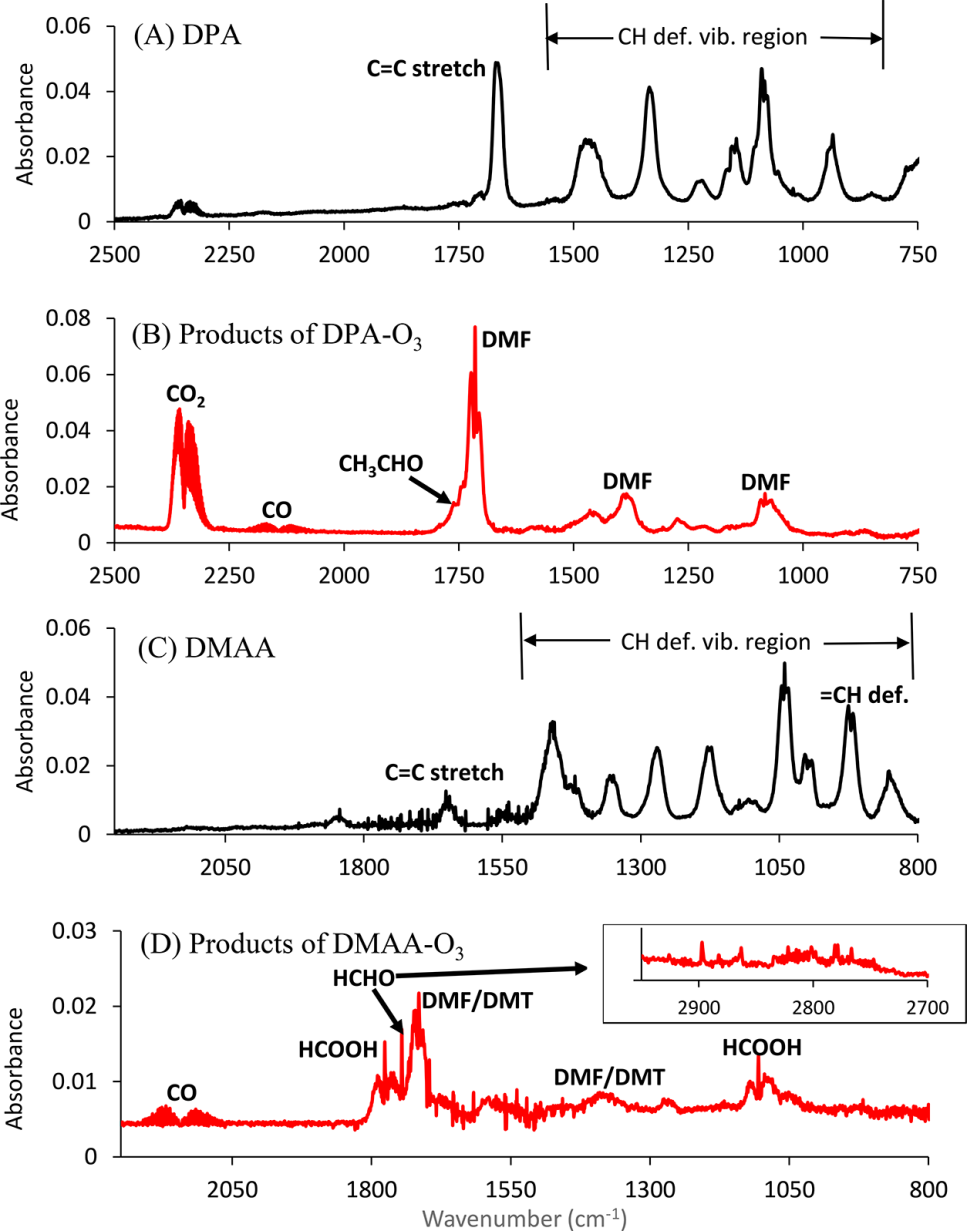
FTIR spectra of (A) DPA, (B) products of DPA ozonolysis after subtraction of the signal from excess DPA, (C) DMAA, and (D) products of DMAA ozonolysis, after subtraction of the signal from excess DMAA.

**Figure 4:**
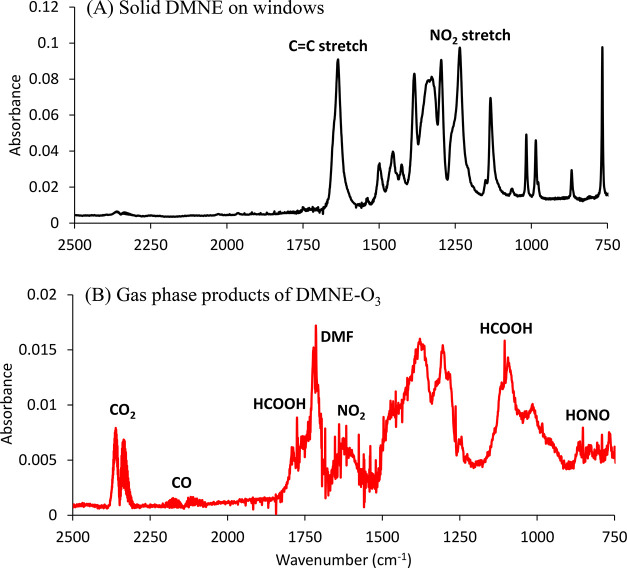
FTIR spectra of (A) solid DMNE on windows and (B) the gas phase products of DMNE ozonolysis after subtraction of the signal from excess DMNE.

Figures 2–4 show the infrared spectra of the gas phase products of ozonolysis of NTP, MNP, DPA, DMAA, and DMNE. These products were identified and quantified by measuring the spectra of authentic samples (CO, CO_2_, NO_2_, dimethylformamide, acetone, and acetaldehyde) or cross sections reported elsewhere for nitrous acid (HONO)^24,25^ as well as formaldehyde (HCHO) and formic acid (HCOOH).^43,27^Table 2 summarizes the product yields expressed as Δ[product]/Δ[O_3_], and the individual product yields of all experiments are reported in Tables S4–S8.

**Table 2:** Product Yields for Gas Phase and Gas–Solid Interface Ozonolysis of Model Compounds^a^^,^^b^

compounds		c	c							
NTP	no C_6_H_12_	0.27 ± 0.09		0.71 ± 0.15	0.18 ± 0.03	0.09 ± 0.02		0.29 ± 0.03	0.10 ± 0.01	
	+C_6_H_12_	0.15 ± 0.02		0.27 ± 0.12	0.13 ± 0.02	0.11 ± 0.01		0.19 ± 0.10	0.11 ± 0.02	
MNP	no C_6_H_12_								0.64 ± 0.03	
	+C_6_H_12_								0.51 ± 0.03	
DPA	no C_6_H_12_		0.50 ± 0.11		0.17 ± 0.02	0.18 ± 0.07				0.46 ± 0.09
	+C_6_H_12_		0.47 ± 0.05		0.14 ± 0.00	0.17 ± 0.04				0.38 ± 0.04
DMAA	no C_6_H_12_		0.15 ± 0.02^d^	0.03 ± 0.01	0.13 ± 0.01				0.33 ± 0.04	
	+C_6_H_12_		0.12 ± 0.02^d^	0.02 ± 0.01	0.09 ± 0.02				0.26 ± 0.02	
DMNE	no C_6_H_12_	0.36 ± 0.07	0.25 ± 0.06	0.31 ± 0.11	0.12 ± 0.04	0.19 ± 0.07	0.02 ± 0.01			
	+C_6_H_12_	0.33 ± 0.04	0.29 ± 0.07	0.26 ± 0.07	0.09 ± 0.02	0.16 ± 0.06	0.02 ± 0.01			

a Errors are 1σ.

b Calculated as the average of product formed divided by the net O_3_ loss at that time except for HONO and DMF.

c Calculated as the maximum HONO or DMF divided by the net O_3_ loss at the time of the maximum HONO or DMF.

d Combination of DMF and DMT.

#### NTP

**Figure 5:**
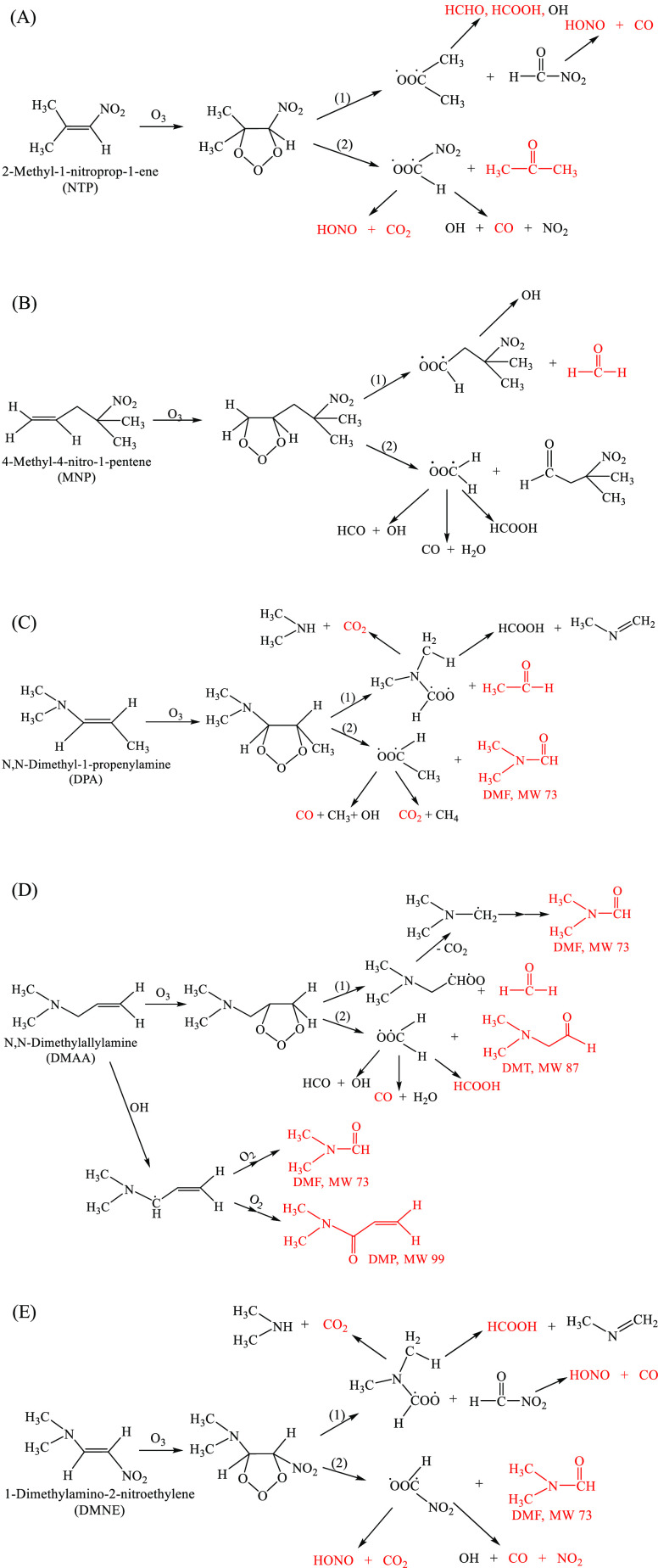
Proposed mechanisms of gas phase ozonolysis of (A) NTP, (B) MNP, (C) DPA, and (D) DMAA and (E) gas–solid interface ozonolysis of DMNE.

The infrared spectrum of NTP is shown in Figure 2A. Products of the gas phase ozone reaction with NTP include formic acid, acetone, HONO, CO, HCHO, and CO_2_ (Figure 2B). Ozone initially adds across the double bond of alkenes, forming a primary ozonide (POZ).^28^ In the gas phase, the POZ decomposes to generate a carbonyl compound and a Criegee intermediate which can further decompose. For asymmetrical alkenes such as NTP, this leads to two sets of initial products as seen in Figure 5A. The first results in the formation of HCOOH, HCHO, HONO, and CO while the second pathway gives HONO, CO_2_, CO, and acetone. Unlike its ozonolysis in solution, the secondary ozonide was not observed here, which is not surprising since the two fragments formed on decomposition of the POZ will be favored and quickly separate in the gas phase.

Addition of cyclohexane as an OH scavenger in the NTP-O_3_ reaction lowered all of the product yields (Table 2), indicating that the OH yield from the ozonolysis of NTP is significant. This is consistent with the reported OH yield of 0.93 from the Criegee intermediate ·OOC(·)(CH_3_)_2_.^29^ Thus, some product formation is likely due to OH chemistry. Formic acid was formed from the ozonolysis of NTP as a major product with yields of 0.71 and 0.27 in the absence and presence of cyclohexane, respectively. Vrbaski and Cvetanovic reported a formic acid yield of 0.22 from the reaction of ozonolysis of 2,3-dimethyl-2-butene (DMB) with high concentrations of the reactants,^30^ while others did not observe formic acid formation from this reaction.^31−26^ To resolve this discrepancy, the ozonolysis of DMB was studied in this system and the results show that formic acid was formed with yields of ∼0.50 and ∼0.19 in the absence and presence of cyclohexane, respectively. This trend is similar to the formic acid yield from NTP ozonolysis. The formation of formaldehyde was likely due to the decomposition of the Criegee intermediate ·OOC(·)(CH_3_)_2_, which was also observed in DMB ozonolysis.^32,26^

#### MNP

Figure 2C shows the spectrum of MNP. Figure 2D shows the infrared spectrum of the gas phase products of MNP ozonolysis. Formaldehyde was observed in high yield, 0.64 ± 0.03, as expected from reaction of a terminal alkene. There was no other carbonyl absorption in the 1700 cm^–1^ region than that due to HCHO at 1745 cm^–1^. An −NO_2_ containing carbonyl product is also expected from the MNP ozonolysis (3-methyl-3-nitrobutanal, Figure 5B) and could further decompose to form other −NO_2_ containing compounds. The 1545 cm^–1^ peak is unidentified but is in the region expected for the asymmetric stretch of a nitro group.

#### DPA

Figure 3A shows the infrared spectrum of DPA. Figure 3B shows the infrared spectrum of the gas phase products of the DPA reaction. When an amino group is present with C=C, the initial O_3_ attack could be on the amine nitrogen and/or C=C double bond.^14^ However, the major organic products observed from DPA ozonolysis were dimethylformamide (DMF) and acetaldehyde (Figure 3B), which were expected from the reaction of O_3_ with the C=C double bond (Figure 5C). There was no direct evidence for attack on the amino group from the nature of the products.

#### DMAA

Figure 3C is the spectrum of DMAA. In DMAA, the amino group is not attached directly to the C=C double bond. The ozonolysis of DMAA also produced a DMF-like carbonyl peak at 1714 cm^–1^ (Figure 3D). Although DMF was not anticipated from O_3_ attack on the C=C directly, a similar compound, 2-(dimethylamino)acetaldehyde (DMT), is expected (Figure 5D). The FTIR spectra of these two compounds should be similar and not easily differentiated.

DART-MS was used to further investigate whether DMF or DMT was formed in the DMAA ozonolysis. Peaks corresponding to [M + H]^+^ for both products at *m*/*z* 74 and 88 were observed (Figure S5), as expected for DMF and DMT, and their MS/MS spectra are shown in Figure S6. A peak at *m*/*z* 147 (MS/MS in Figure S6) was identified as the dimer of DMF, which might be generated inside the mass spectrometer. Another product, *N*,*N*-dimethyl-2-propenamide (DMP) at *m*/*z* 100 can be formed by abstraction of an allylic hydrogen from the −CH_2_– group by OH radicals, followed by addition of O_2_ and secondary reactions of the RO_2_ that is formed. There was a small but consistent decrease in the product yields in the presence of the cyclohexane scavenger (Table 2), indicating that decomposition of the Criegee intermediates generated some OH (second pathway for POZ decomposition in Figure 5D) that could then attack DMAA to form DMP. Formaldehyde and formic acid were also observed from DMAA ozonolysis (Figure 3B). DMF is proposed to form from O_3_ attack on C=C followed by the decomposition of the Criegee intermediate from pathway 1 in Figure 5D. It can also be formed from initial abstraction of an allylic hydrogen in a pathway parallel to that forming DMP (Figure 5D).

#### DMNE

Figure 4A is the spectrum of solid DMNE on the cell windows. In DMNE, both nitro and amino groups are attached to the double bond. Ozonolysis in the liquid phase (CCl_4_) showed that O_3_ preferentially attacked the C=C.^14^Figure 4B shows a typical FTIR spectrum of products of DMNE-O_3_ reaction after about 10 min reaction time. No products were observed after the gas phase was pumped out, confirming all products were in the gas phase.

Time profiles for O_3_ and the products HCOOH, HONO, DMF, NO_2_, CO_2_, and CO are shown in Figure S7 for a typical experiment. These products are consistent with the initial O_3_ attack on C=C, similar to the DMNE ozonolysis in CCl_4_ solution.^14^ Nitrous acid and DMF slowly decrease when the ozone is consumed, likely due to loss to the cell walls. Table S8 summarizes the product yields expressed as Δ[product]/Δ[O_3_]. The product yields were similar in the presence or absence of the OH scavenger cyclohexane,^17^ suggesting that the OH yield from decomposition of a Criegee intermediate in the DMNE ozonolysis is small. The formation of formic acid was attributed to the decomposition of Criegee intermediate ·OOCH(·)N(CH_3_)_2_. However, *N*-methylmethanimine (CH_2_=NCH_3_) was not observed, possibly due to a low IR absorption cross-section. The proposed reaction scheme is shown in Figure 5E.

It is noteworthy that none of the three compounds that have amino groups, DPA, DMAA, and DMNE, shows evidence in their products for attack of ozone at the amino group. This is in contrast to the reactions in CCl_4_^14^ where the ozonolysis products from DPA and DMAA indicated there was attack at the amino group. However, this difference in gas vs solution phase is consistent with kinetics measurements of the reactions of tertiary amines with ozone in the two phases. For example, the rate constant for O_3_ with trimethylamine in solution is 4.1 × 10^6^ M^–1^ s^–1^,^34^ much faster than the rate constant, ∼10^5^ M^–1^ s^–1^, for terminal alkenes in solution.^35^ On the other hand, Tuazon et al.^36^ measured a room temperature rate constant for O_3_ with trimethylamine of 7.8 × 10^–18^ cm^3^ molecule^–1^ s^–1^, similar to typical values of ∼10^–17^ cm^3^ molecule^–1^ s^–1^ for reaction of O_3_ with terminal alkenes.^28^ However, there was no direct evidence from the products identified here for a significant contribution from attack on the amine.

### Environmental Significance

Since amino, nitro, and C=C groups are widely used in emerging contaminants such as pharmaceuticals, pesticides, and munitions, it is crucial to understand their synergistic effects on the kinetics and mechanisms. It is clear that in both the gas and liquid phases the nitro group attached to the C=C significantly decreases the alkene ozonolysis reaction rate constants, with gas phase lifetimes of the nitro alkenes with respect to 100 ppb O_3_ of 134 and 7 days, respectively, for NTP and MNP. An amino group has the opposite effect, with lifetimes for DPA and DMAA shorter than 5 days.

The presence of both groups in DMNE provides counterbalancing effects. However, this is more complicated in that it is a gas–solid reaction that occurs both at the surface and in the bulk. From the KM-GAP model, the surface rate constant is 1.8 × 10^–9^ cm^2^ s^–1^, and at a gas phase O_3_ concentration of 100 ppb, the surface O_3_ concentration is 2.2 × 10^7^ cm^–2^. This gives a lifetime for the surface DMNE of only 25 s. Using the partitioning coefficient for O_3_ of 4 × 10^–4^ mol cm^–3^ atm^–1^ (Table S3) and the bulk phase rate constant of 1.0 × 10^–16^ cm^3^ s^–1^, a lifetime of 7 min is estimated. This is a lower limit since the bulk concentration of ozone will decrease due to reaction and diffusion in the layers. At any rate, the lifetime of DMNE should be quite short compared to the other model compounds.

Attack at either or both the amine nitrogen and the double bond occurs in solution depending on the structure, but in the gas phase, reaction of ozone with the double bond predominates. Nitrous acid (HONO) from the decomposition of the Criegee intermediate ·OOCH(·)NO_2_ was a common product in all media. This is an important species affecting the oxidant balance in the atmosphere, and in the liquid phase, it further reacts with secondary and tertiary amines to generate toxic *n*-nitroso compounds.^37−39^ These insights provide key data for assessing the environmental fates of emerging contaminants with amine, alkene, and/or nitro groups which exist in all phases and on environmental surfaces.

## Supporting Information Available

Experimental apparatus, reactions and rate constants used in Kintecus and the fitting results, details of quantitative modeling of multiphase DMNE ozonolysis and the fitting results, and product yields for all individual experiments (PDF)
