# Supplementary material for: Gas Phase and Gas–Solid Interface Ozonolysis of Nitrogen Containing Alkenes: Nitroalkenes, Enamines, and Nitroenamines
Source: J Phys Chem A. 2022 Aug 4;126(32):5398–406. doi: 10.1021/acs.jpca.2c04400 (PMC9393864; doi:10.1021/acs.jpca.2c04400)
Supplement: Supplementary file 1 — jp2c04400_si_001.pdf [file jp2c04400_si_001.pdf]

Supporting Information

Gas Phase and Gas-Solid Interface Ozonolysis of Nitrogen Containing Alkenes: Nitroalkenes, Enamines, and Nitroenamines

Weihong Wang,[§a] Xinke Wang,[§a] Pascale S. J. Lakey,[a] Michael J. Ezell,[a] Manabu Shiraiwa[a] and Barbara J. Finlayson-Pitts[*a]

[a] Department of Chemistry, University of California, Irvine, CA 92697-2025

[§]These authors contributed equally to this work.

[*]**Email:** bjfinlay@uci.edu

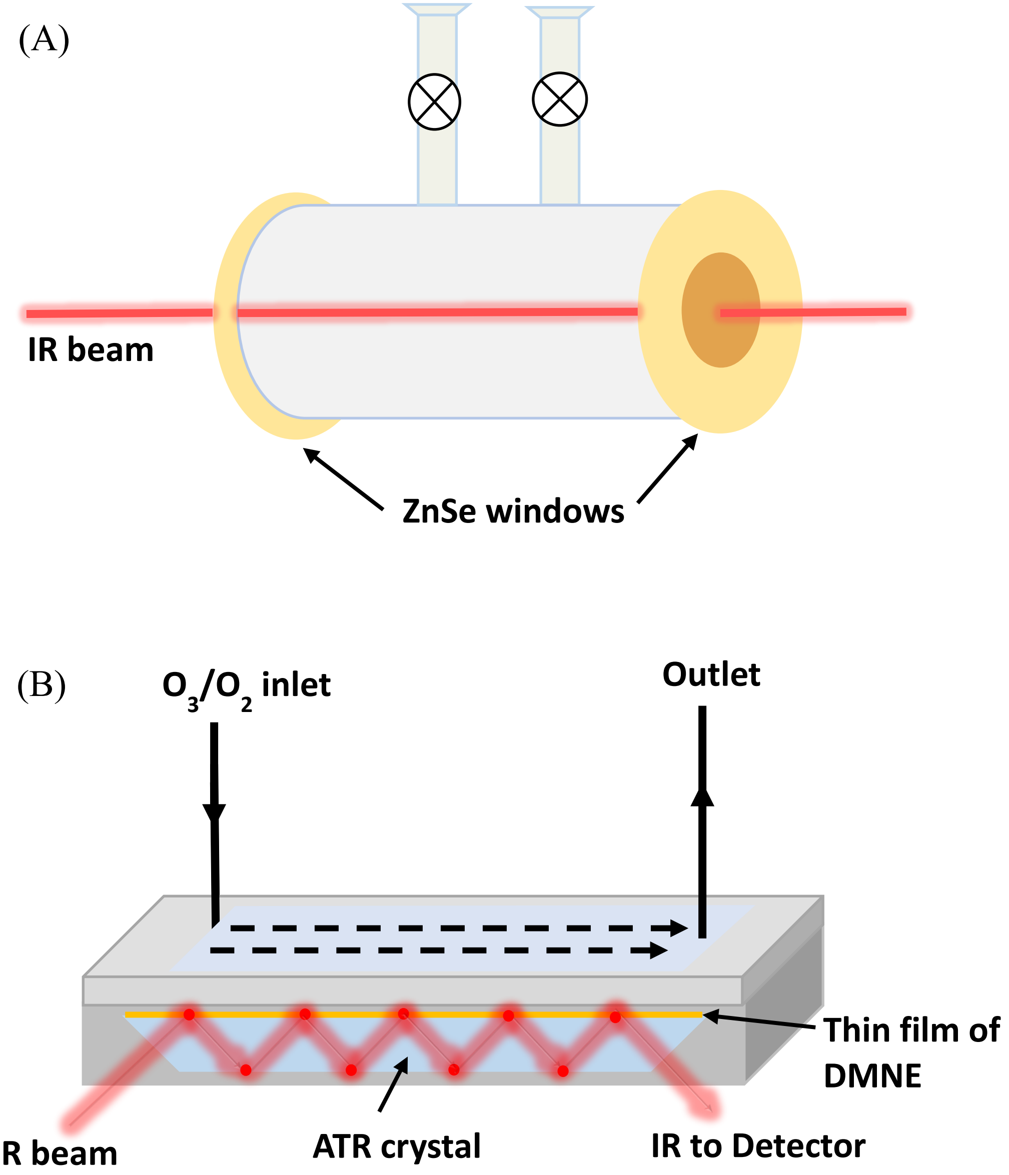

Figure S1. (A) Transmission cell used for gas phase kinetics and product studies, and (B) ATR cell used for kinetics studies of gas-solid DMNE ozonolysis.

Table S1. Reactions and rate constants for NTP ozonolysis.[a]

Reactions	***k***[b,c]
O_3 + NTP ==> C_2H_6COO + $HCONO_2$	k_1[d]
O_3 + NTP ==> $OOCH(NO_2)$ + CH_3COCH_3	k_2[d]
C_2H_6COO ==> OH + HCOOH	1.40E+02[e]
C_2H_6COO ==> OH + HCHO	2.00E+01[e]
$OOCH(NO_2)$ ==> HONO + CO_2	1.00E+02[e]
$OOCH(NO_2)$ ==> OH + CO + NO_2	1.00E+01[e]
OH + HCOOH ==> products	4.00E-13
OH + HCHO ==> HCO + H_2O	8.50E-12
NTP ==> wall	9.00E-05[e]
HONO ==> wall	4.50E-04[e]
HONO ==> $HONO_{(ads)}$	3.50E-04[e]
$HONO_{(ads)}$ + $HONO_{(ads)}$ ==> NO_2 + NO + H_2O	1.00E-17[f]
NO_2 + H_2O ==> $HONO_{(ads)}$	2.40E-23[f]
NO_2 + H_2O ==> $HNO_{3(ads)}$	2.40E-23[f]
N_2O_5 ==> $HNO_{3(ads)}$ + $HNO_{3(ads)}$	2.00E+02[f]
$HONO_{(ads)}$ + H_2O ==> HONO + H_2O	1.35E-20[f]
$HONO_{(ads)}$ + $HNO_{3(ads)}$ ==> NO_2 + NO_2	1.00E-17[f]
NO + NO + O_2 ==> NO_2 + NO_2	2.00E-38
O_3 + HONO ==> O_2 + HNO_3	5.00E-19
OH + CH_3COCH_3 ==> products	2.20E-13[g]
OH + NTP ==> CH_3COCH_3	1.00E-13[e]
OH + NTP ==> products	1.00E-13[e]
OH + CO ==> HO_2 + CO_2	2.40E-13
O + O_2 ==> O_3	1.50E-14
O + NO_2 ==> NO + O_2	1.00E-11
O + O_3 ==> O_2 + O_2	8.00E-15
O + NO ==> NO_2	1.60E-12
O_3 + NO ==> NO_2 + O_2	1.90E-14
O_3 + NO_2 ==> NO_3 + O_2	3.20E-17
O_3 + $HCONO_2$ ==> HONO + CO_2 + O_2	5.00E-19[e]
$HCONO_2$ ==> HONO + CO	2.00E+02[e]
NO_3 + NO_2 ==> N_2O_5	1.20E-12
NO_3 + NO_2 ==> NO + NO_2	6.60E-16
NO + NO_3 ==> NO_2 + NO_2	2.60E-11
N_2O_5 ==> NO_3 + NO_2	4.00E-02
NO_3 + NO ==> NO_2 + NO_2	2.60E-11
O + NO_3 ==> NO_2 + O_2	1.00E-11
NO_3 + NO_3 ==> NO_2 + NO_2	2.30E-16
O + NO_2 ==> NO_3	3.30E-12
HO_2 + NO ==> OH + NO_2	8.10E-12
OH +NO_2 ==> HNO_3	1.00E-11

$OH + O_3$ ==> $HO_2 + O_2$	7.30E-14
$HO_2 + O_3$ ==> OH	1.90E-15
$HO_2 + HO_2$ ==> $H_2O_2 + O_2$	5.90E-12
$OH + H_2O_2$ ==> $HO_2 + H_2O$	1.70E-12
OH + NO ==> HONO	7.30E-12
OH + OH ==> H_2O + O	1.80E-12
OH + OH ==> H_2O_2	6.30E-12
$OH + HO_2$ ==> $H_2O + O_2$	1.10E-10
O + OH ==> O_2 + H	3.30E-11
$O + HO_2$ ==> $OH + O_2$	5.90E-11
$O + H_2O_2$ ==> $OH + HO_2$	1.70E-15
$H + O_2$ ==> HO_2	9.20E-13
$H + O_3$ ==> $OH + O_2$	2.90E-11
$OH + NO_2$ ==> HOONO	1.80E-12
HOONO ==> $OH + NO_2$	8.18E-01
$HO_2 + NO_2$ ==> HO_2NO_2	1.10E-12
HO_2NO_2 ==> $HO_2 + NO_2$	6.90E-02
$H + HO_2$ ==> OH + OH	7.20E-11
$H + HO_2$ ==> $O + H_2O$	1.60E-12
$H + HO_2$ ==> $H_2 + O_2$	6.90E-12
$OH + H_2$ ==> H_2O + H	6.70E-15
$O + N_2O_5$ ==> products	3.06E-16
$O + HNO_3$ ==> $OH + NO_3$	3.00E-17
$O + HO_2NO_2$ ==> products	8.60E-16
$H + NO_2$ ==> OH + NO	1.30E-10
$OH + NO_3$ ==> $HO_2 + NO_2$	2.20E-11
OH + HONO ==> $H_2O + NO_2$	4.50E-12
$OH + HNO_3$ ==> $H_2O + NO_3$	1.50E-13
$OH + HO_2NO_2$ ==> products	4.60E-12
$HO_2 + NO_3$ ==> $OH + NO_2$	3.50E-12
$OH + C_6H_{12}$ ==> $C_6H_{10}O$	7.20E-12[g]

[a]Rate equations integrated using Kintecus.[4]
[b]The units for k are s^{-1} for first order reaction, cm^3 $molecule^{-1}$ s^{-1} for second order reactions and cm^6 $molecule^{-2}$ s^{-1} for third order reactions, respectively.
[c]The values are from JPL Publication 19-5 except for those noted.[5]
[d]The rate constants for all experiments are listed in Table S4. The branching ratio for k_1/k_{total} for O_3 + NTP varied from 0.43 – 0.63.
[e]Best fit values from the experimental measurements.
[f]From Ramazan et al.[6]
[g]From Finlayson-Pitts and Pitts.[7]

Table S2. Reactions and rate constants for MNP ozonolysis.[a]

Reactions	k[b,c]
O_3 + MNP ==> HCHO + CI	k_1[d]
O_3 + MNP ==> $\cdot OOC(\cdot)H_2{*}$ + $HCOC_4H_8NO_2$	k_2[d]
CI ==> OH + products	1.00E+02[e]
CI ==> products	1.00E+01[e]
$\cdot OOC(\cdot)H_2{*}$ ==> HCO + OH	1.20E+01[f]
$\cdot OOC(\cdot)H_2{*}$ ==> CO + H_2O	3.80E+01[f]
$\cdot OOC(\cdot)H_2{*}$ ==> CO_2 + H_2	3.30E+01[f]
$\cdot OOC(\cdot)H_2{*}$ ==> $\cdot OOC(\cdot)H_2$	1.70E+01[f]
$\cdot OOC(\cdot)H_2$ + H_2O ==> HCOOH + H_2O	2.80E-16
$\cdot OOC(\cdot)H_2$ + $\cdot OOC(\cdot)H_2$ ==> products	7.12E-11
$\cdot OOC(\cdot)H_2$ + HCOOH ==> products	1.10E-10
OH + MNP ==> HCHO + $HCOC_4H_8NO_2$	3.00E-11[e]
OH + HCHO ==> HCO + H_2O	8.50E-12
OH + $HCOC_4H_8NO_2$ ==> products	3.00E-11[e]
OH + CO ==> HO_2 + CO_2	2.40E-13
OH + HCO ==> H_2O + CO	1.80E-10
O_3 + HCO ==> H + O_2 + CO_2	8.30E-13
O_2 + HCO ==> HO_2 + CO	5.20E-12
OH + O_3 ==> HO_2 + O_2	7.30E-14
HO_2 + O_3 ==> OH + O_2 + O_2	1.90E-15
HO_2 + HO_2 ==> H_2O_2 + O_2	5.90E-12
OH + H_2O_2 ==> HO_2 + H_2O	1.70E-12
OH + OH ==> H_2O + O	1.80E-12
OH + OH ==> H_2O_2	6.30E-12
OH + HO_2 ==> H_2O + O_2	1.10E-10
OH + H_2 ==> H_2O + H	6.70E-15
O + HCHO ==> HCO + OH	1.60E-13
O + OH ==> O_2 + H	3.30E-11
O + HO_2 ==> OH + O_2	5.90E-11
O + H_2O_2 ==> OH + HO_2	1.70E-15
O + O_2 ==> O_3	1.50E-14
O + O_3 ==> O_2 + O_2	8.00E-15
H + O_2 ==> HO_2	1.15E-12
H + O_3 ==> OH + O_2	2.90E-11
H + HO_2 ==> OH + OH	7.20E-11
H + HO_2 ==> O + H_2O	1.60E-12
H + HO_2 ==> H_2 + O_2	6.90E-12
OH + C_6H_{12} ==> $C_6H_{10}O$	7.20E-12[g]

[a]Rate equations integrated using Kintecus.[4]

[b]The units for k are s^{-1} for first order reactions and cm^3 $molecule^{-1}$ s^{-1} for second order reactions, respectively.

[c]The values are from JPL Publication 19-5 except for those noted.[5]

[d]The rate constants for all experiments are listed in Table S5. The branching ratio for $k_1/(k_1+k_2)$ for O_3 + MNP varied from 0.43 – 0.50.

[e]Best fit values from the experimental measurements.

[f]Branching ratio for $\cdot OOC(\cdot)H_2$* is from Finlayson-Pitts and Pitts.[7]

[g]From Finlayson-Pitts and Pitts.[7]

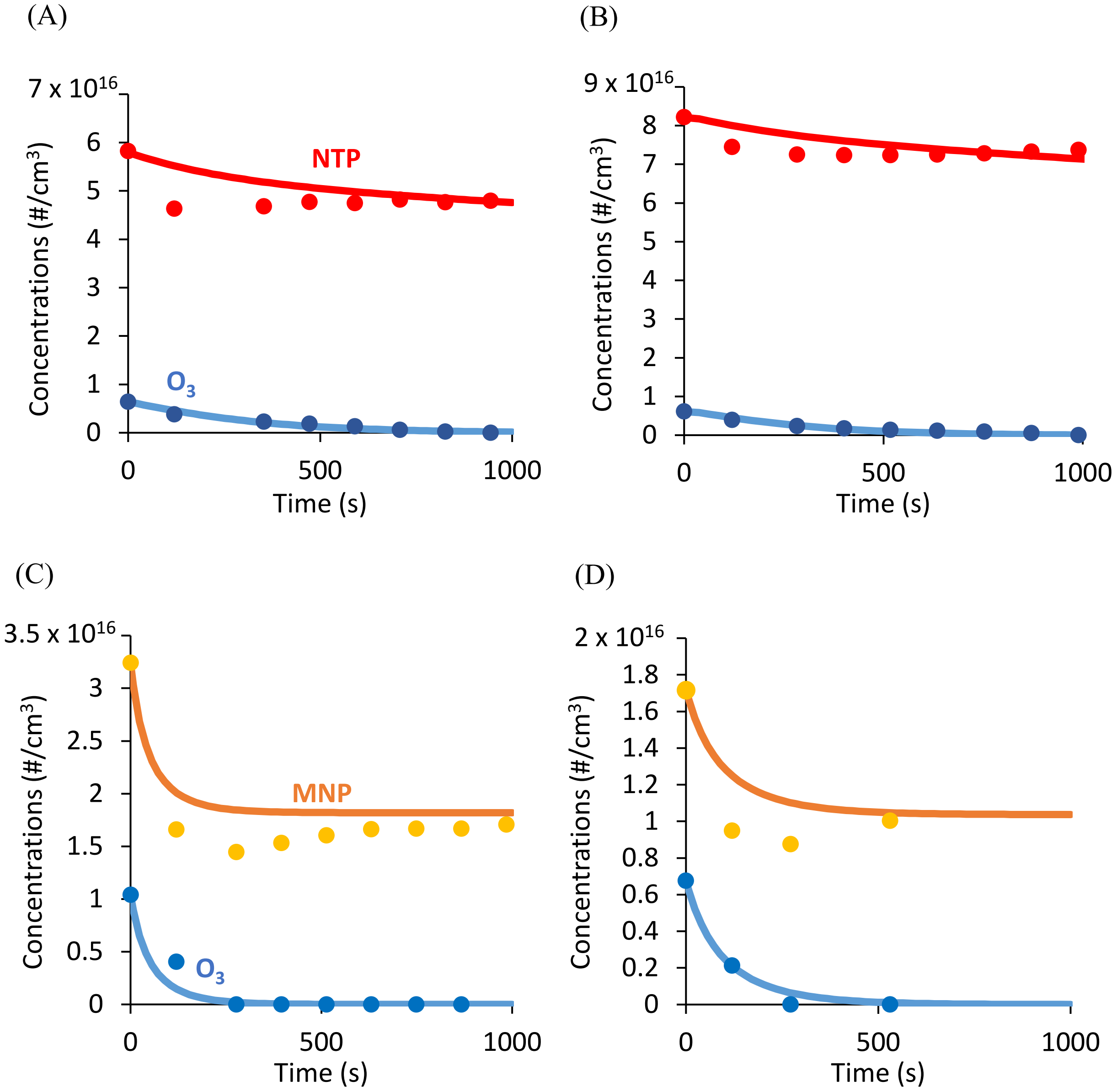

Figure S2. Experimental measurements (closed circles) and the best fit (lines) using the reactions in Tables S1 for NTP ozonolysis (A) in the absence of cyclohexane and (B) in the presence of cyclohexane, and using the reactions in Tables S2 for MNP ozonolysis (C) in the absence of cyclohexane and (D) in the presence of cyclohexane.

Text S1. Quantitative modeling of multi-phase DMNE ozonolysis.

A modified version of the kinetic multi-layer model of gas-particle interactions in aerosols and clouds (KM-GAP)[1] was used to better understand the loss of DMNE on the crystal surface as a function of time, film thickness and ozone concentration. Briefly, processes included in KM-GAP are gas-phase diffusion, reversible adsorption to a surface, partitioning into a solid, bulk diffusion, and a chemical reaction. The reaction of DMNE with ozone is included in the film bulk at the surface and in the gas phase. As molecules diffuse into or out of a bulk layer, the layer will grow or shrink accordingly. This version of the KM-GAP model is modified to allow bulk layers to split once their thickness reaches more than two monolayers or merge once layers reach a thickness of less than 0.5 monolayers as described in Lakey et al.[2] This allows high resolution and short diffusion lengths to be maintained in the film without layers becoming unrealistically large or small. The gas phase was treated using the following differential equation:

$$\frac{\mathrm{d[X]_g}}{\mathrm{dt}} = \left([\mathrm{X}]_{\mathrm{g,0}} - [\mathrm{X}]_{\mathrm{g}}\right)\frac{F}{V} + \frac{D_{\mathrm{g,X}}}{mfp_{\mathrm{X}}}\left([\mathrm{X}]_{\mathrm{gs}} - [\mathrm{X}]_{\mathrm{g}}\right)\frac{S}{V} + L_{\mathrm{g,X}} \qquad \text{(Eq. 1)}$$

where $[\mathrm{X}]_{\mathrm{g,0}}$, $[\mathrm{X}]_{\mathrm{g}}$ and $[\mathrm{X}]_{\mathrm{gs}}$ are the gas phase concentration of species X entering the reaction chamber at t =0, and the bulk phase and near-surface concentrations respectively at time t. *F* is the flow rate through the chamber, *V* is the volume of the chamber, *S* is the surface area of the crystal, $D_{\mathrm{g,X}}$ is the gas phase diffusion coefficient of species X, mfp_{X} is the mean free path of species X and $L_{\mathrm{g,X}}$ is the gas phase loss rate due to chemical reactions. Parameters used in the model are summarized in Table S3 and were either fixed based on literature values or determined by fitting to the experimental data. The saturation vapor pressure of DMNE obtained from best fit of the model is used in the first order surface to bulk mass transport term as described in the original KM-GAP paper.[3] An assumption was made that all products were extremely volatile and partitioned to the gas phase instantly except for one non-volatile product which was assumed to have a mass equal to that of DMNE combined with ozone.

Table S3. Parameters used in the KM-GAP model

**Parameter**	**Parameter description**	**Value**	**Reference or comment**
S (cm^2)	Surface area of the crystal	4	Experimental value
V (cm^3)	Volume of the chamber	2	Experimental value
F (lpm)	Flow rate	1 (unless specifically stated)	Experimental value
$[O_3]_{g,0}$ (ppb)	Ozone concentration entering the reaction chamber	140 – 950	
$\alpha_{s,0}$	Surface mass accommodation of all species at time 0	1	
$\tau_{d,all}$ (s)	Desorption lifetime of all species from the surface	1×10^{-9}	
P_{DMNE} (Torr)	Saturation pressure of DMNE	4×10^{-5}	Determined by fitting to the data
D_{DMNE} (cm^2 s^{-1})	Diffusion coefficient of DMNE in the bulk	5×10^{-17}	Determined by fitting to the data
$D_{product}$ (cm^2 s^{-1})	Diffusion coefficient of the non-volatile product in the bulk	5×10^{-17}	Assumed to be the same as D_{DMNE}
D_{O3} (cm^2 s^{-1})	Diffusion coefficient of ozone in the bulk	1×10^{-8}	Determined by fitting to the data
$D_{g,DMNE}$ (cm^2 s^{-1})	Gas phase diffusion coefficient of DMNE	0.08	Determined using the EPA calculator
$D_{g,O3}$ (cm^2 s^{-1})	Gas phase diffusion coefficient of ozone	0.16	Ref. 8
K_{O3} (mol cm^{-3} atm^{-1})	Partitioning coefficient of ozone	4×10^{-4}	Consistent with ozone partitioning into other organics under dry conditions[9]
k_{br} (cm^3 s^{-1})	Bulk rate coefficient of ozone with DMNE	4×10^{-17} (no product)<br>1×10^{-16} (product)	Determined by fitting to the data
k_s (cm^2 s^{-1})	Surface rate coefficient of ozone with DMNE	7.1×10^{-10} (no product)<br>1.8×10^{-9} (product)	Assumed to be equal to k_{br}/ thickness of 1 DMNE molecule Note that the surface reaction occurs between adsorbed ozone and adsorbed DMNE and also

			adsorbed ozone and DMNE in bulk layer 1.
k_g (cm^3 s^{-1})	Gas phase rate coefficient of ozone with DMNE	0	Sensitivity tests showed that the rapid removal of DMNE from the gas phase meant that this reaction could not compete. In the final model the rate was set to 0.
k_{wall} (s^{-1})	First order loss rate of DMNE to the chamber walls	1 (Figure S3 panel (g))<br>0.7 (All other figures)	Determined by fitting to the data
$Y_{product}$	Yield of the non-volatile product from the reaction of DMNE with ozone	0.06	Determined by fitting to the data

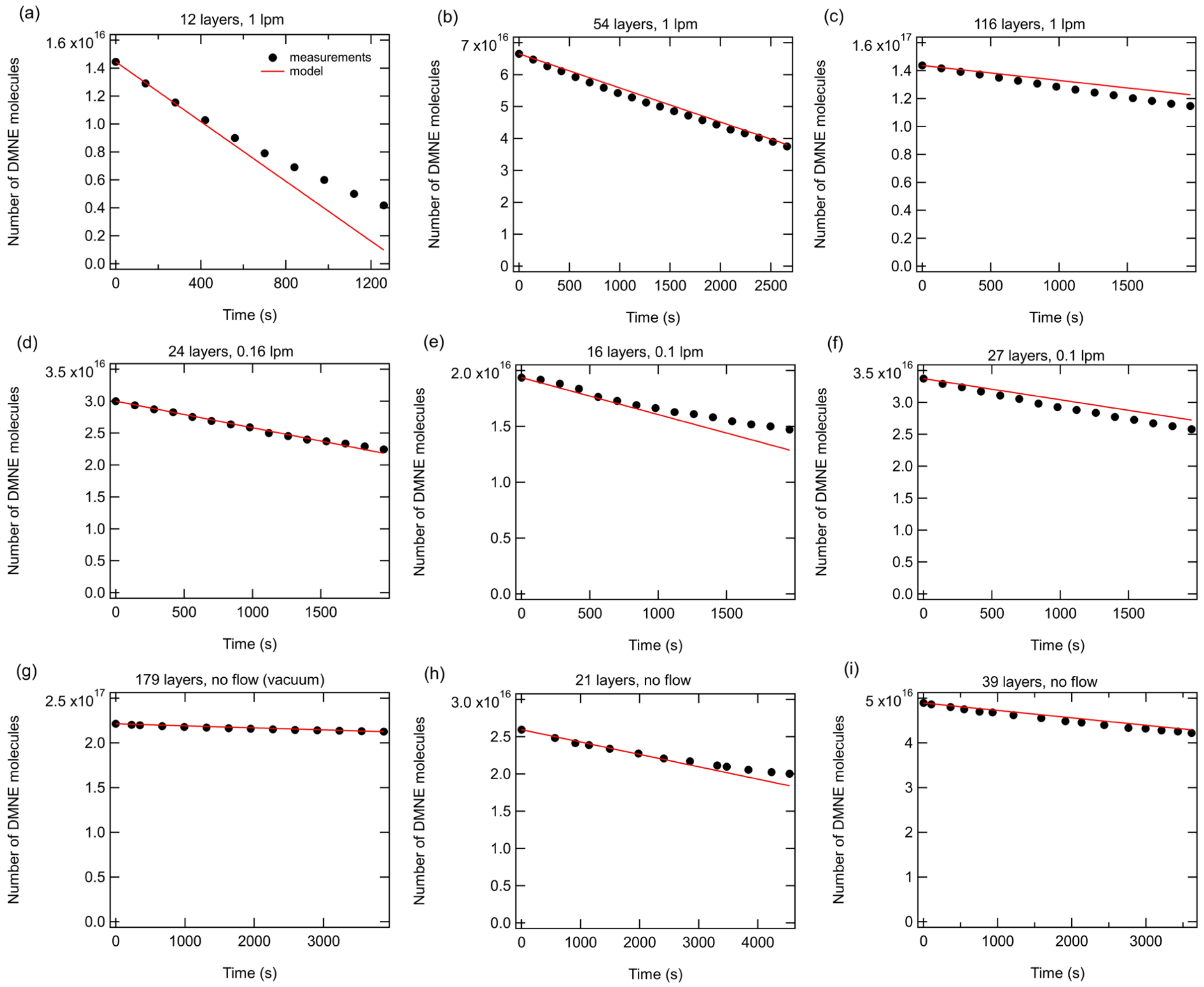

Figure S3. Decay of DMNE (black dots) from the crystal surface in the absence of ozone with different numbers of DMNE monolayers and flow rates of N_2.

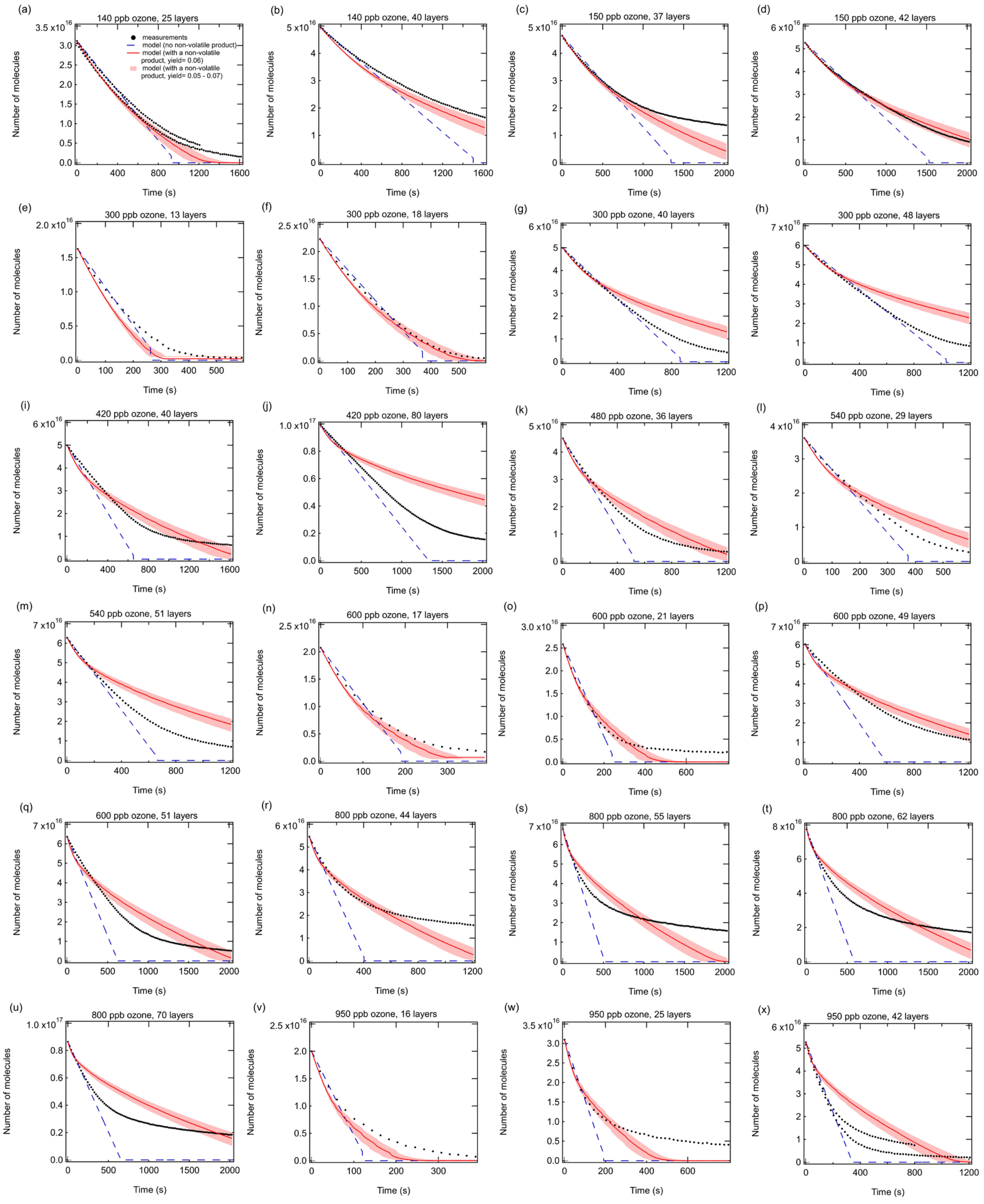

Figure S4. Decay of DMNE from the crystal surface in the presence of different ozone concentrations and with different numbers of DMNE monolayers. Dashed blue lines are for model runs where all products are assumed to be volatile and instantly partition to the gas phase while red lines are model runs where a non-volatile product is formed with a yield of 0.06. The pink shading represents DMNE molecules when the yield of the non-volatile product is varied from 0.05 – 0.07. There are two sets of experimental data in (a) and (x).

Table S4. Product yields and rate constants for gas phase NTP ozonolysis.

Initial NTP concentrations (× 10^{16} cm^{-3})	Initial ozone concentrations (× 10^{15} cm^{-3})	Initial C_6H_{12} concentrations (× 10^{17} cm^{-3})	k (× 10^{-20} cm^3 $molecule^{-1}$ s^{-1})[a]	$\frac{\Delta Acetone}{\Delta O_3}$	$\frac{\Delta HCOOH}{\Delta O_3}$	$\frac{\Delta HCHO}{\Delta O_3}$	$\frac{\Delta CO}{\Delta O_3}$	$\frac{\Delta CO_2}{\Delta O_3}$	$\frac{\Delta HONO^b}{\Delta O_3}$
5.8	6.4	--	4.0	0.32	0.79	0.10	0.18	0.06	0.19
4.7	6.1	--	4.0		0.80	0.12	0.12		0.24
3.5	6.3	--	2.8	0.28	0.91		0.15	0.10	0.42
2.7	10.0	--	5.0	0.26	0.75	0.10	0.20	0.09	
3.1	8.9	--	3.2	0.27	0.80	0.10	0.22		
6.8	8.9	--	3.5	0.34	0.46	0.11	0.22		0.20
4.1	5.8	--	4.7	0.27	0.62	0.11	0.18	0.12	0.32
7.4	6.3	--	2.2	0.27	0.59	0.09	0.17	0.10	0.23
		**Average**	**3.9**	**0.29**	**0.71**	**0.10**	**0.18**	**0.09**	**0.27**
		σ	0.8	0.03	0.15	0.01	0.03	0.02	0.09
3.3	8.1	6.5	2.2	0.24	0.44	0.10			
3.6	5.7	8.1	3.0	0.35	0.33	0.09	0.16	0.12	0.16
8.2	6.2	8.7	3.2	0.14	0.14	0.13	0.13		0.16
7.4	6.5	7.4	4.0	0.13	0.20	0.11	0.12		0.13
8.0	6.3	8.7	3.2	0.10	0.22	0.13	0.11	0.11	0.17
		**Average**	**3.1**	**0.19**	**0.27**	**0.11**	**0.13**	**0.11**	**0.15**
		σ	0.6	0.10	0.12	0.02	0.02	0.01	0.02
		**Total average for *k***	**3.5**						
		σ	0.9						

[a]Determined by fitting the experimental data using the reaction scheme in Table S1.
[b]Calculated as the maximum HONO divided by the net O_3 loss at the time of the maximum HONO.

Table S5. Product yields and rate constants for gas phase MNP ozonolysis.

**Initial MNP concentrations ($\times 10^{16}$ cm^{-3})**	**Initial ozone concentrations ($\times 10^{15}$ cm^{-3})**	**Initial C_6H_{12} concentrations ($\times 10^{17}$ cm^{-3})**	***k* ($\times 10^{-19}$ cm^3 molecule^{-1} s^{-1})[a]**	**$\frac{\Delta HCHO}{\Delta O_3}$**
3.2	10.4	--	6.5	0.67
1.6	9.2	--	7.0	0.63
0.9	9.2	--	5.5	0.59
1.6	6.8	--	7.0	0.64
1.0	5.9	--	7.5	0.64
4.5	6.9	--	6.0	0.64
		**Average**	**6.6**	**0.64**
		σ	0.7	0.03
1.0	7.9	6.5	8.0	0.49
1.7	6.8	6.5	6.5	0.47
1.3	8.8	5.8	7.5	0.52
0.9	11.0	7.1	6.5	0.54
		**Average**	**7.1**	**0.51**
		σ	0.8	0.03
		**Total average for *k***	**6.8**	
		σ	0.8	

[a]Determined by fitting the experimental data using the reaction scheme in Table S2.

Table S6. Product yields for gas phase DPA ozonolysis.

Initial DPA concentrations ($\times 10^{16}$ cm^{-3})	Initial ozone concentrations ($\times 10^{16}$ cm^{-3})	Initial C_6H_{12} concentrations ($\times 10^{18}$ cm^{-3})	$\frac{\Delta DMF^{a}}{\Delta O_3}$	$\frac{\Delta CO}{\Delta O_3}$	$\frac{\Delta CO_2}{\Delta O_3}$	$\frac{\Delta CH_3CHO}{\Delta O_3}$
3.2	1.1	--	0.48	0.17	0.19	0.47
1.7	1.0	--	0.36	0.14	0.14	0.37
3.7	0.7	--	0.59	0.18	0.13	0.54
		**Average**	**0.47**	**0.16**	**0.15**	**0.46**
		σ	0.12	0.02	0.03	0.09
3.5	1.1	3.1	0.50	0.14	0.13	0.38
1.8	1.1	2.7	0.41	0.14	0.13	0.35
3.6	0.7	3.2	0.50	0.14	0.22	0.42
		**Average**	**0.47**	**0.14**	**0.16**	**0.38**
		σ	0.05	0.00	0.05	0.04

[a]Calculated as the maximum DMF divided by the net O_3 loss at the time of the maximum DMF.

Table S7. Product yields for gas phase DMAA ozonolysis.

Initial DMAA concentrations (× 10^{16} cm^{-3})	Initial ozone concentrations (× 10^{15} cm^{-3})	Initial C_6H_{12} concentrations (× 10^{17} cm^{-3})	$\frac{\Delta DMF^{a}}{\Delta O_3}$	$\frac{\Delta HCOOH}{\Delta O_3}$	$\frac{\Delta HCHO}{\Delta O_3}$	$\frac{\Delta CO}{\Delta O_3}$
3.7	7.4	--	0.19	0.01	0.31	0.10
2.4	7.8	--	0.15	0.02	0.31	0.12
0.8	8.1	--	0.14	0.03	0.31	0.12
0.5	9.6	--	0.12	0.03	0.30	0.13
0.8	9.4	--	0.14	0.03	0.30	0.13
0.7	9.9	--	0.14	0.03	0.37	0.14
0.6	9.3	--	0.13	0.04	0.38	0.14
0.9	9.1	--	0.16	0.03	0.40	0.14
		**Average**	**0.15**	**0.03**	**0.33**	**0.13**
		σ	0.02	0.01	0.04	0.01
0.8	11.0	4.0	0.10	0.02	0.26	0.08
0.6	10.0	3.9	0.12	0.03	0.30	0.08
2.8	9.9	6.8	0.14	0.01	0.25	0.12
1.7	8.3	7.0	0.12	0.02	0.25	0.07
3.5	7.8	7.2	0.13	0.01	0.25	0.10
		**Average**	**0.12**	**0.02**	**0.26**	**0.09**
		σ	0.02	0.01	0.02	0.02

[a]Calculated as the maximum DMF divided by the net O_3 loss at the time of the maximum DMF.

Table S8. Yields of gas phase products in the reaction of O_3 with thin films of DMNE on ZnSe windows.

Total number of DMNE molecules on windows ($\times 10^{17}$ molecules)	Total number of ozone added ($\times 10^{17}$ molecules)	Total number of C_6H_{12} added ($\times 10^{18}$ molecules)	$\frac{\Delta DMF^a}{\Delta O_3}$	$\frac{\Delta HCOOH}{\Delta O_3}$	$\frac{\Delta CO}{\Delta O_3}$	$\frac{\Delta CO_2}{\Delta O_3}$	$\frac{\Delta HONO^a}{\Delta O_3}$	$\frac{\Delta NO_2}{\Delta O_3}$
3.6	3.3	--	0.21	0.36	0.12		0.29	0.03
2.1	2.9	--	0.25	0.45	0.18		0.33	0.01
2.1	3.1	--	0.19	0.43	0.11		0.33	0.01
4.1	3.5	--	0.21	0.37	0.08		0.33	0.02
3.3	3.2	--	0.20	0.40	0.11	0.16	0.30	0.02
2.1	2.1	--	0.21	0.44	0.13		0.38	0.02
2.4	5.1	--	0.22	0.34	0.11		0.32	
3.1	4.8	--	0.21	0.42	0.13		0.38	
3.6	2.2	--	0.17	0.22	0.08		0.28	0.04
2.4	2.3	--	0.21	0.22	0.09		0.25	0.02
3.4	3.7	--	0.24	0.28	0.08	0.12	0.27	0.02
1.0	3.5	--	0.31	0.12	0.11	0.18	0.38	
1.8	3.4	--	0.39	0.28	0.12	0.14	0.47	0.01
3.0	1.9	--	0.38	0.21	0.14	0.23	0.44	0.01
3.4	2.3	--	0.29	0.16	0.10	0.20	0.46	
0.4	2.1	--	0.26	0.21	0.21	0.32	0.47	
		**Average**	**0.25**	**0.31**	**0.12**	**0.19**	**0.36**	**0.02**
		σ	0.06	0.11	0.04	0.07	0.07	0.01
3.3	3.6	4.6	0.24	0.42	0.14		0.38	0.02
3.8	2.1	3.2	0.19	0.25	0.09		0.36	0.04
2.4	2.3	2.6	0.28	0.25	0.09		0.34	0.03
3.6	3.4	4.4	0.26	0.27	0.07	0.13	0.28	0.02
3.4	3.5	5.1	0.26	0.26	0.08	0.14	0.29	0.03
2.0	3.6	5.1	0.29	0.17	0.08	0.10	0.33	
3.0	2.1	4.2	0.38	0.20	0.11	0.24	0.37	0.01
3.6	2.4	4.5	0.39	0.26	0.09	0.20	0.32	0.01
		**Average**	**0.29**	**0.26**	**0.09**	**0.16**	**0.33**	**0.02**
		σ	0.07	0.07	0.02	0.06	0.04	0.01

[a]Calculated as the maximum HONO or DMF divided by the net O_3 loss at the time of the maximum HONO or DMF.

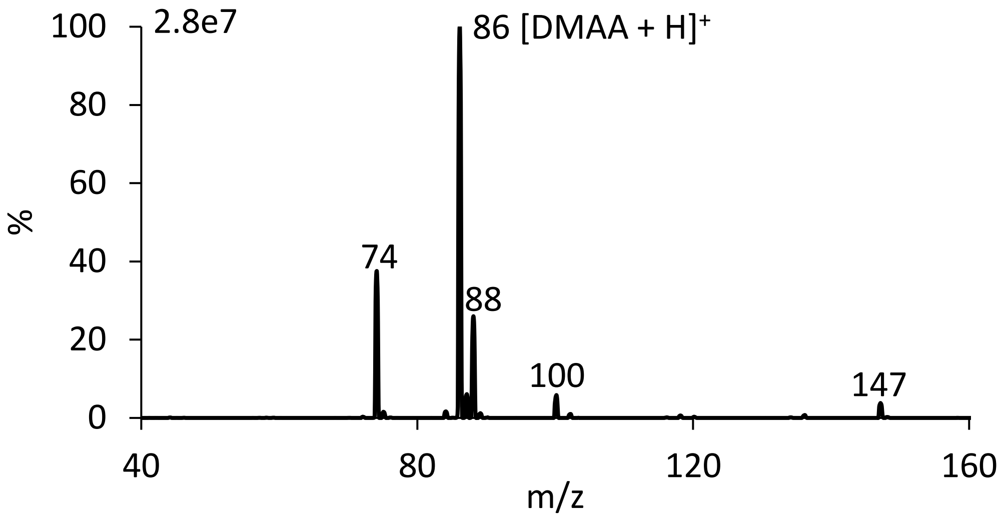

Figure S5. DART-MS of DMAA after ozonolysis.

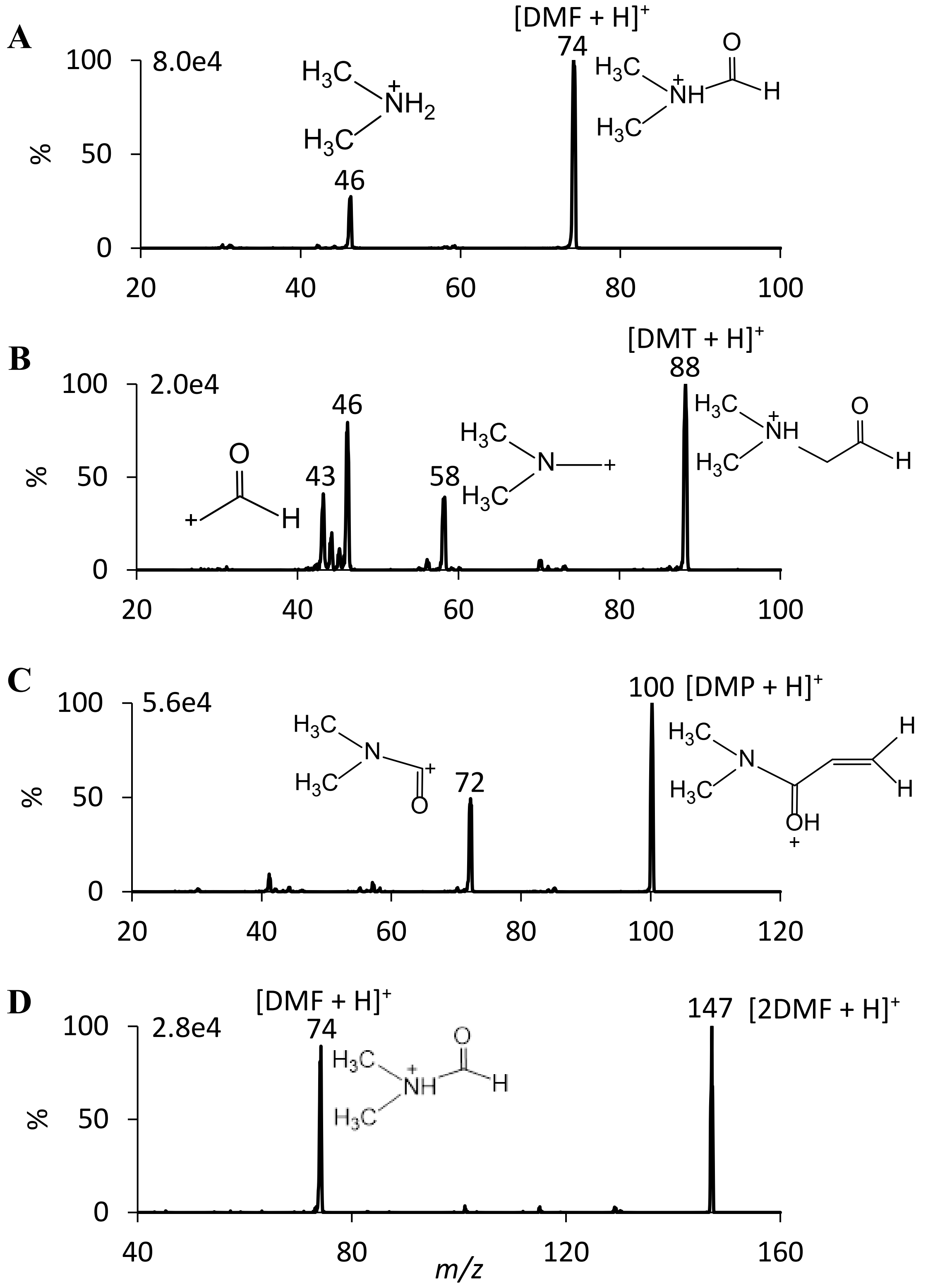

Figure S6. DART-MS/MS of products of DMAA ozonolysis at *m/z* (A) 74, (B) 88, (C) 100 and (D) 147.

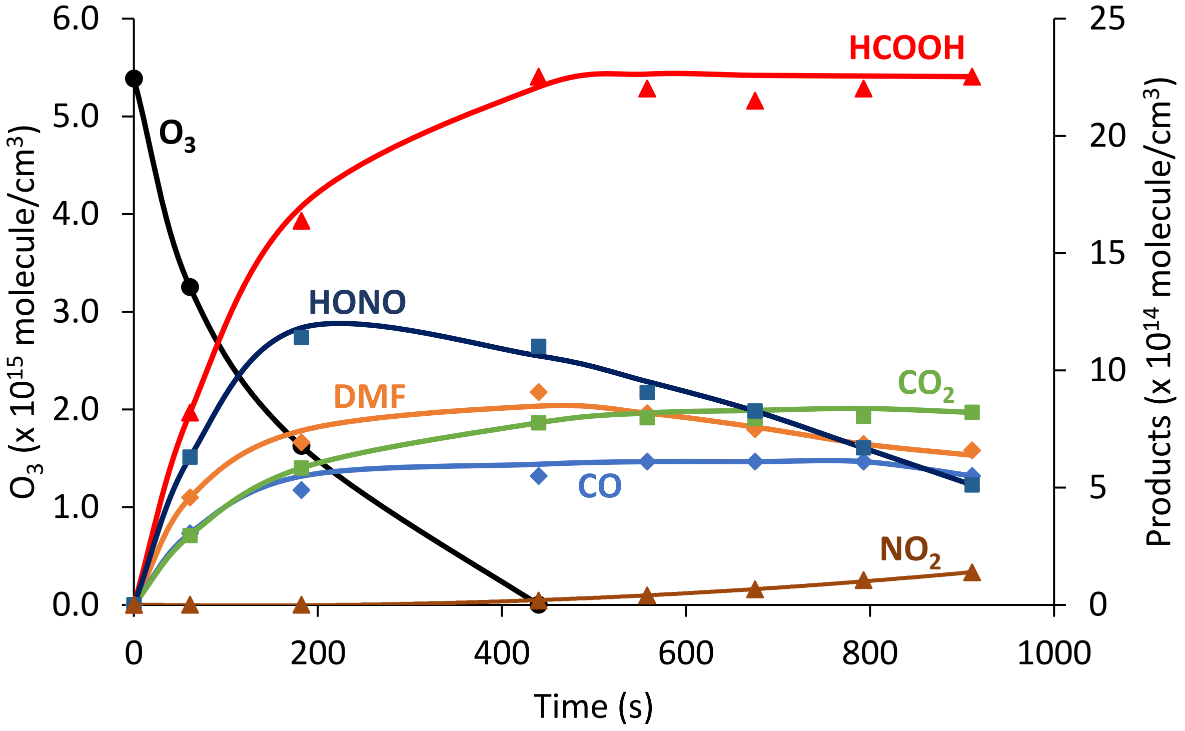

Figure S7. Typical time profile of loss of ozone and formation of products during DMNE ozonolysis. Lines are a guide to the eye.

References

(1) Shiraiwa, M.; Pfrang, C.; Koop, T.; Pöschl, U. Kinetic Multi-Layer Model of Gas-Particle Interactions in Aerosols and Clouds (KM-GAP): Linking Condensation, Evaporation and Chemical Reactions of Organics, Oxidants and Water. *Atmos. Chem. Phys.* **2012**, *12* (5), 2777–2794. https://doi.org/10.5194/acp-12-2777-2012.

(2) Lakey, P. S. J.; Eichler, C. M. A.; Wang, C.; Little, J. C.; Shiraiwa, M. Kinetic Multi-Layer Model of Film Formation, Growth, and Chemistry (KM-FILM): Boundary Layer Processes, Multi-Layer Adsorption, Bulk Diffusion, and Heterogeneous Reactions. *Indoor Air* **2021**, *31* (6), 2070–2083. https://doi.org/10.1111/ina.12854.

(3) Wang, W.; Ezell, M. J.; Lakey, P. S. J.; Aregahegn, K. Z.; Shiraiwa, M.; Finlayson-Pitts, B. J. Unexpected Formation of Oxygen-Free Products and Nitrous Acid from the Ozonolysis of the Neonicotinoid Nitenpyram. *Proc. Natl. Acad. Sci. U. S. A.* **2020**, *117* (21), 11321–11327. https://doi.org/10.1073/pnas.2002397117.

(4) Ianni, J. C. A Comparison of the Bader-Deuflhard and the Cash-Karp Runge-Kutta Integrators for the GRI-MECH 3.0 Model Based on the Chemical Kinetics Code Kintecus. In *Computational fluid and solid mechanics 2003*; Bathe, K. J., Ed.; Elsevier Science Ltd.: Oxford, U.K., 2003; pp 1368–1372.

(5) Burkholder, J. B.; Sander, S. P.; Abbatt, J. P. D.; Barker, J. R.; Huie, R. E.; Kolb, C. E.; Kurylo, M. J.; Orkin, V. L.; Wilmouth, D. M.; Wine, P. H. Chemical Kinetics and Photochemical Data for Use in Atmospheric Studies, Evaluation No. 19. *JPL Publ. 19-5* **2020**, No. 19, 1–153.

(6) Ramazan, K. A.; Syomin, D.; Finlayson-Pitts, B. J. The Photochemical Production of HONO during the Heterogeneous Hydrolysis of NO_2. *Phys. Chem. Chem. Phys.* **2004**, *6* (14), 3836. https://doi.org/10.1039/b402195a.

(7) Finlayson-Pitts, B. J.; Pitts Jr, J. N. *Chemistry of the Upper and Lower Atmosphere: Theory, Experiments, and Applications*; Academic Press: San Diego, CA, 2000.

(8) Massman, W. J. A Review of the Molecular Diffusivities of H_2O, CO_2, CH_4, CO, O_3, SO_2, NH_3, N_2O, NO, and NO_2 in Air, O_2 and N_2 near STP. *Atmos. Environ.* **1998**, *32* (6), 1111–1127. https://doi.org/10.1016/S1352-2310(97)00391-9.

(9) Berkemeier, T.; Steimer, S. S.; Krieger, U. K.; Peter, T.; Pöschl, U.; Ammann, M.; Shiraiwa, M. Ozone Uptake on Glassy, Semi-Solid and Liquid Organic Matter and the Role of Reactive Oxygen Intermediates in Atmospheric Aerosol Chemistry. *Phys. Chem. Chem. Phys.* **2016**, *18* (18), 12662–12674. https://doi.org/10.1039/c6cp00634e.
